# Mechanism properties of a bird-neck bionic rigid-flexible structure

**DOI:** 10.1016/j.fmre.2022.06.023

**Published:** 2022-08-06

**Authors:** Xiuting Sun, Jian Xu, Zhifeng Qi

**Affiliations:** School of Aerospace Engineering and Applied Mechanics, Tongji University, Shanghai 200092, China

**Keywords:** Bionic structure, Rigid-flexible structure, Spatial combination deformation, Integral form, Nonlinear restoring force

## Abstract

By the biological construction of a bird neck, a bionic bird-neck multilevel rigid-flexible structure is proposed and some biometric properties are explained. The proposed structure can flexibly deform in six directions, which inspires the study of its mechanical properties for flexible deformations. First, the structural configuration and composition are determined based on the study of the anatomical characteristics of the woodpeckers. Since the skeletons and muscles have very different values for the elasticity modulus and the deformation is mostly dependent on the muscle tension, the bionic structure consists of rigid units and bio-syncretic components. For combined deformations, the mechanical model is established by the connectivity matrix to describe the connection of each level. Second, based on the principle of minimum potential energy, an integral form-finding method is proposed for flexible combination deformations. All of the integral forms obtained with the theoretical analysis are compared with the results with Finite Element Analysis. The structural parameters of the bionic structure were then tightly fixed to the actual shape of the bird's neck and the corresponding overall form took on an "S" shape, which perfectly matched the construction of the bird's neck. In addition, for the pre-deformation form, by analyzing the potential energy of the bionic structure, due to the adjustable dynamic stiffness property, an explanation is provided for the significant dynamic stability of the bird neck in bending. This study not only proposes a bionic rigid-flexible structure with high spatial accessibility but also explains biological properties of a bird neck based on the study of its mechanics characteristics. Based on the modeling and the mechanical properties of the bionic structure in flexible spatial combination deformations, the multi-steady state, and the variable dynamic stiffness, the bird-neck bionic rigid-flexible structure has significant applications such as aeronautical deployable systems, manipulator positioning, and dynamic stability fields.

## Graphical Abstract

**Figure:**
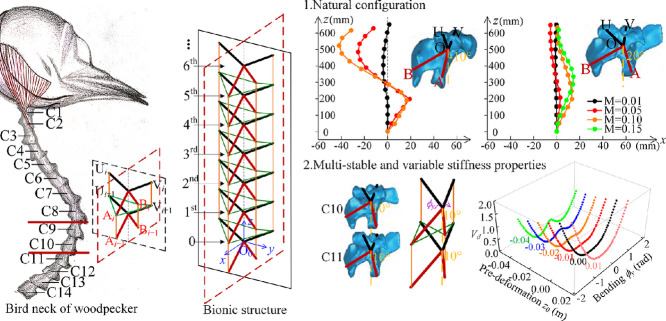

## 1 Introduction

The skeletal system and the muscular system are the vital organ systems of a vertebrate. The skeletal system supports the body, protects the internal organs, provides muscle attachment, and acts as a lever for movement. The muscular system is attached to the skeleton and is innervated by motor nerves, which enables the body of an animal to perform various actions through its own contractile properties. The analysis of the construction and behaviors of the musculoskeletal system can aid in understanding the working principles and optimal design of variable bionic structures [1]. For the corresponding bionic structure in vertebrates, the typical model includes multiple rigid-flexible coupling systems [2], which are widely utilized in the fields of mechanical engineering, aerospace engineering [3,4], and robotics [5,6], among others. Since the biological system of a bird neck is the result of evolution, the corresponding bionic structures can achieve powerful functions with the optimization of the biological interpretability. Therefore, sufficient studies on the construction as well as the static and dynamic behaviors of the skeletal and muscular systems are required.

In the field of mechanical engineering structures and robotics, one of the most important functions is the designed spatial deformation and motion. To achieve the required spatial accessibility, different flexible mechanisms for bionic structures have been proposed. In order to achieve rapid expansion, Zareei et al. [7] provided a cascade architecture that was bio-inspired by the maniphalanx and could be activated by an initial signal based on a bistable mechanism design. The maniphalanx-inspired cascade mechanism could produce bending deformation in the horizontal plane and display a C integral form. In order to assist a paralytic to recover finger grasping ability, a limb exoskeleton system was designed and actuated by an artificial muscle to induce dynamic bending movements [8,9]. For mechanical series arms, stability is an unsolved issue, while it is difficult for parallel arms to achieve large stroke operations [10,11]. Based on active control methods, the designed mechanical arms can complete a tracking function with favorable stability. For the bionics of dynamic processes, an octopus arm-inspired flexible bending structure made with soft materials and soft drives [12], [13], [14] was proposed to achieve the stable and precise grasping of objects with different shapes. In summary, for bio-inspired musculoskeletal systems such as limb and maniphalanx systems, the rigid units in the corresponding bionic structures are connected by hinges or joints that cooperate with elastic components. The most frequent deformation of the existing bionic structures is bending, while torsion deformation is relatively difficult to implement. In addition, due to the connections such as hinges and joints, the friction affects the movement behaviors and positioning accuracy [15].

In nature, birds have no periocular muscles to position their view, and thus, without the stabilization and spatial accessibility ability of their necks, the shaky view induced by flapping and walking greatly affects the vision of a bird, which increases the difficulties in holding gazes and avoiding obstacles. Navigating airspace and controlling a flight path based on visual stabilization are the typical roles of the bird-neck structure [16,17]. Utilizing high-speed videos, Quinn et al. [18,19] designed a bio-experiment for a whooper swan and obtained the head-to-body motion transfer function in flight over a lake. The dynamic behaviors of the head and body of an avian have been previously studied [20]. The motions of the neck and head of birds during the whole process of walking can be divided into two parts: holding and thrusting [21]. As the definition states, for holding, the body moves from an original position to the next one, and the head stays in the same position. For thrusting, the body has a slight gentle movement, while the head thrusts to the next position, relying on the deformation of the neck structure. For terminal orientation, the neck structure changes the integral form to coordinate the movements of the body and the head. As is well-known, the neck structure has large torsion deformation ability based on the coordinated actuation of different groups of muscles. Therefore, for bionic mechanisms or structures to satisfy the requirement for the elimination of friction and torsion accessibility, the structural morphology and function implementation are the two most important features. By observing the structures of bird necks, the most apparent structural feature is the connection of multiple skeletons by muscles.

It should be noted that the rigidity properties of skeletons and muscle groups in necks are entirely different. In some previous studies, tensegrity structures were noticed that contained rigid-body units and tensioning ropes. Since a tensegrity structure has lightweight and flexible characteristics, these types of structures are appropriate for imitating the structural morphology, and they have potential applications for the function implementations of foraging, flying stabilization, and bearing head weight, as for bird neck [22]. Recently, due to the deformation reachability and designability of the tensegrity structures, they have been utilized to imitate a manipulator arm for acquiring space targets [23]. Several tensegrity structures bio-inspired by bird necks [24], snake spines [25,26], and fish fins [27] have been proposed. In the analysis of the modeling of a bird-neck bionic tensegrity structure, a connectivity matrix is defined to describe the connection of each rigid unit, imitating the action of the muscles. Based on an X-shaped structure [28,29], the mechanical properties such as the equilibrium and the workspace of the bird-neck bionic tensegrity structure have been analyzed. For the actions of force systems, integral form-finding methods have been proposed based on the static equilibrium method [30], dynamic relaxation method [31], and Monte-Carlo method [32]. Furthermore, for the bionic arm tensegrity mechanism, control methods have been proposed for the accuracy of the orientation acquisition [33].

**Fig. 1:**
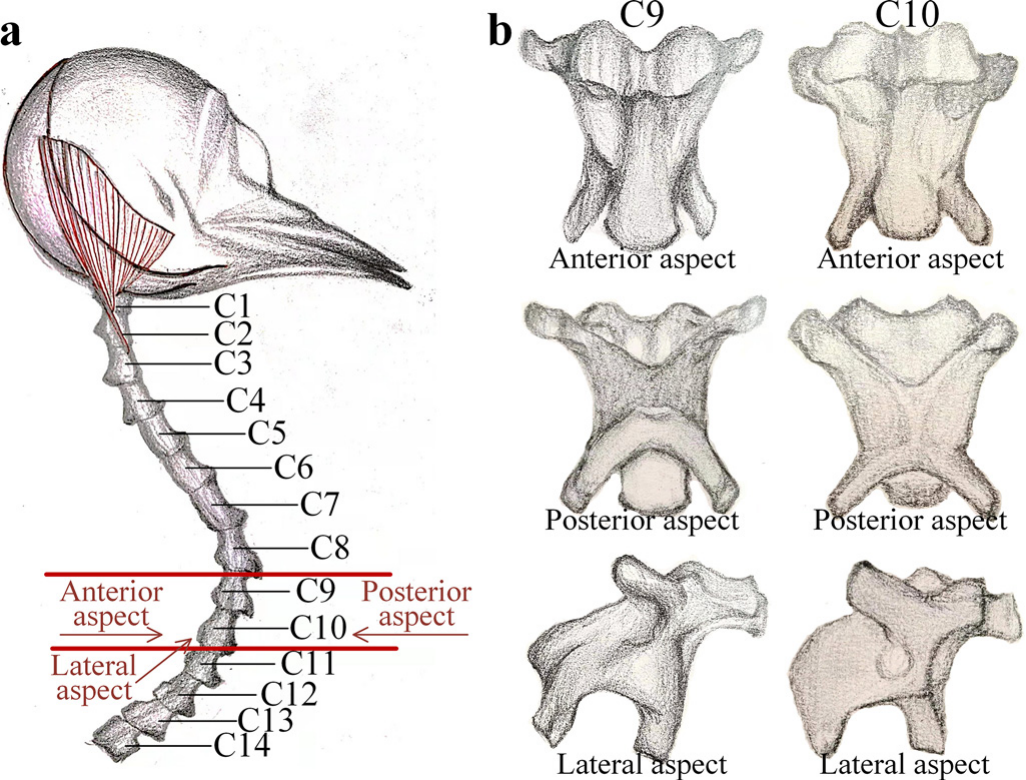
**The biological anatomy diagram of a woodpecker neck.** (a) Skeleton configuration of the bird neck; (b) shape of skeletons C9 and C10. (Referring to the anatomical structure of a woodpecker in Ref. [34], drawn by SUN X.T.). C*_i_* (*i* = 1,2,…,14) is the segment number for the skeleton; three angles of view are given as the anterior, posterior and lateral aspects to show the geometry configuration of one section of the skeleton.

In a bionic structure, the rigid units imitate the skeletons and the bio-syncretic components imitate the muscles to provide the tension forces. The configuration of the rigid unit and the connection form of the bio-syncretic components are the key factors. A clear bionic mechanism can improve the accurate modeling of the bionic structure and broaden the scope of its applications. The bird neck structure and skeletons C9-C10 are shown in Fig. 1. Based on the anatomical map of the skeleton system of a woodpecker [34], it was discovered that there were 14 sections of skeletons in its neck structure connected by different groups of muscles. According to the anatomy results [35], the shapes of the skeletons and connections of the muscles in the neck structure had similar configurations. Additionally, the skeletons are connected by muscle groups, mainly including the interspinales muscles, upper neck muscles, long nape muscles, and anterior cervical ligament [34,35]. The muscle groups have the effect of stretching and tensioning, and they connect a skeleton. Based on the coordination forces by muscle groups, the neck structure can bear the weight located in the specified position and stabilize the head motion. Thus, the proposed bird-neck bionic structure is a multilevel rigid-flexible coupling structure with isomorphic rigid units, and it can flexibly deform in space.

In this study, several areas of study are undertaken to propose a novel bird-neck bionic structure with high spatial accessibility including torsion deformation. First, to provide accurate mechanical modeling for large combination deformations, the bionic mechanism of the multilevel rigid-flexible coupling structure has to be clearly analyzed, including the shape of the rigid units and the connection of the bio-syncretic components. Second, for an arbitrary force system, a fast calculation method for integral form-finding is proposed to lay the study foundation for stability control. Then, the mechanical properties of the bird-neck bionic structure can be used to explain the functions of bird necks with respect to the natural configuration and dynamic stabilization in vibration isolations [36,37]. In the FEA modeling process, the configurations and elasticity modulus of the material are set according to refs. [38], [39], [40]. The rest of this paper is organized as follows. The rigid-flexible coupling structure inspired by a bird neck is proposed and the corresponding model is established in Section 2. Then, the integral form-finding method for the arbitrary force system is presented and several study cases are provided in Section 3. The displacements and deformations of elastic components are discussed for bearing weight on a slant to explain the loading capacity of the multilevel bionic structure. The mechanical properties presented in Section 4 show the possibility of the adjustable nonlinear stiffness property of the proposed bionic structure. Finally, the conclusions are presented.

## 2 Bionic rigid-flexible structure

### 2.1 Introduction of the bird neck

As shown in Fig. 1, the most obvious construction feature of a bird neck is the coupling of the two components of the skeletons and muscles. Since the skeletons and muscles have very different values for the elasticity modulus, in the bionic structure, the skeletons are considered to be rigid units and the muscles are considered to be bio-syncretic components. Then, the shape of the rigid units and the connection type of the bio-syncretic components in the proposed bionic structure are the key points.

**Fig. 2:**
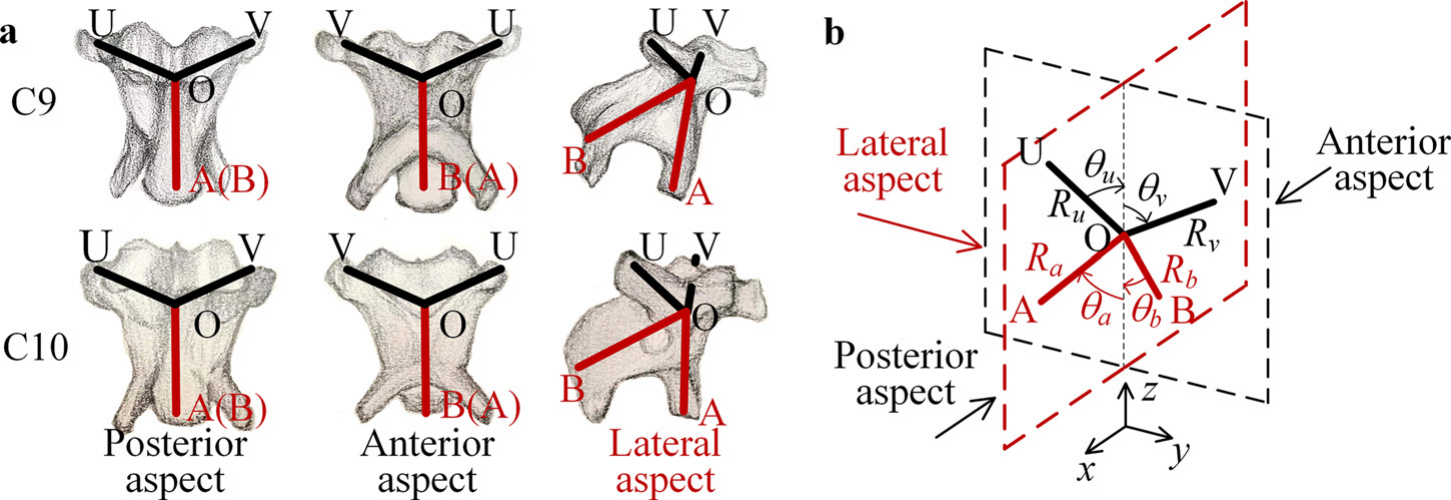
**The shape of the skeletons and the corresponding rigid body.** (a) Shape of the skeletons C9 and C10 in a bird neck and the catapophysis points for the muscles. (b) One simplified rigid unit in the bionic structure. ((a) refers to the anatomical structure of a woodpecker in Ref. [34], drawn by SUN X.T.). In the tortile “X”-shaped rigid unit, the center is O; The symbols A, B, U and V are the catapophysis of each skeleton. The intersection angles between the two bottom rods to the vertical axis are *θ_a_* and *θ_b_*, and the corresponding angles for the upper rods are *θ_u_* and *θ_v_*; The lengths of the two bottom rods are *R_a_* and *R_b_*, and the lengths of the upper rods are *R_u_* and *R_v_*.

According to the observation of the skeletons in the bird neck, as shown in Fig. 1, for one skeleton, the catapophysis points are the key connecting points for the muscles to combine each skeleton into a slender neck structure. As shown in Fig. 2a, the distribution and the location of the side catapophysis points are symmetrical in the posterior and anterior aspects. In the lateral aspect, there are front and rear catapophysis points. Therefore, the configuration of the rigid part of the skeleton is a spatial structure with multiple rods simulating catapophysis. As shown in Fig. 2b, the center of the skeleton is the point O. For the posterior aspect, the upper two sides of catapophysis are in the plane *yoz*, which are the right point U and the left point V. For the lateral aspect, the bottom two catapophyses are in the plane *xoz*, including the front catapophysis point A and the rear catapophysis point B. According to the shape of one level of the skeleton, as shown in Fig. 2a, it is simplified as a rigid unit with the shape and structural parameters shown in Fig. 2b, which is a tortile “X” with the four catapophysis points U, V, A, and B.

Based on the observation and analysis of the shape of each skeleton in the bird neck structure, we consider that the skeletons have similar shapes. According to the shapes of the skeletons, as shown in Fig. 2a, each rigid unit in the proposed bionic structure has the shape of a tortile “X,” as shown in Fig. 2b.

**Fig. 3:**
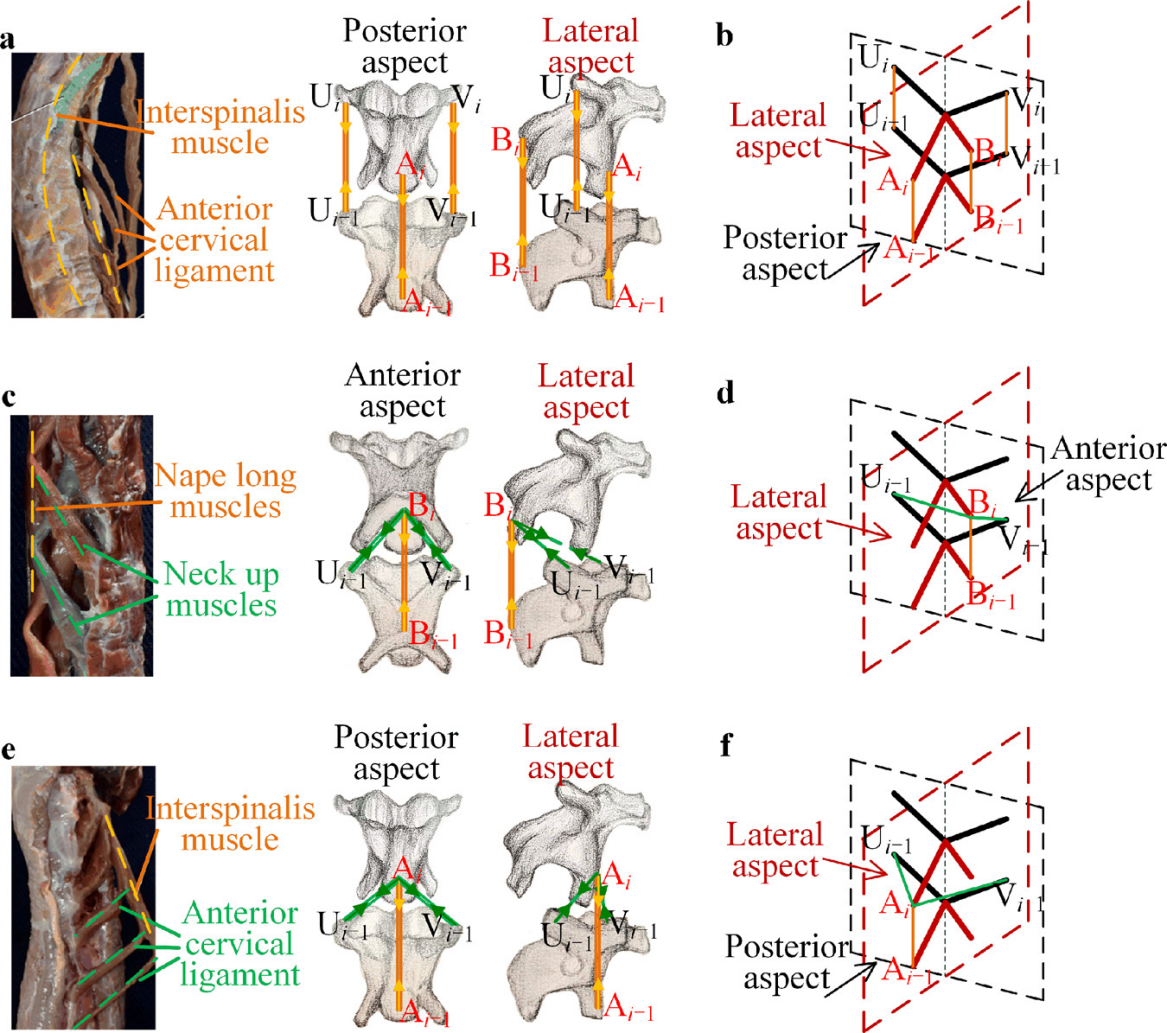
**The muscle groups in the bird neck and the corresponding connections.** (a) Interspinalis muscle connection at the catapophysis, and (b) the corresponding bio-syncretic components; (c) nape muscle group, including the interspinalis muscle, upper neck muscles, and the long nape muscles, and (d) the corresponding bio-syncretic components; (e) neck abdominal muscle group, including the interspinalis muscle and anterior cervical ligament, and (f) the corresponding bio-syncretic components. (Referring to the anatomical structure of a woodpecker in Refs. [34], [35], drawn by SUN X.T.)

After determining the shape of the rigid units by studying the key points of skeletons with respect to the neck structure, the other key feature in the bird neck is the connection of the muscles among the different levels of the skeletons. The bio-syncretic components in the bionic structure are utilized to describe the connection points and the attendant modes of the muscle groups. Thus, it is necessary to study the connection and action of different group muscles. Fig. 3 shows the different muscle groups, their connections among the skeletons, and the forces acting on each skeleton, which determine the arrangement of the bio-syncretic components in the bionic structure to connect the rigid units.

From the bio-experiment and the observation of the neck structure, the most important muscle group is the interspinalis muscle on the front and rear sides. As shown in Fig. 3a, in the posterior and lateral aspects, the orange lines represent the interspinalis muscles connected to the same catapophysis of the two skeletons along the vertical direction, as shown in the force lines. According to the interspinalis muscles shown in Fig. 3a, in the proposed bionic structure, four vertical bio-syncretic components are assembled at the end of each rod to connect the rigid units, as shown in Fig. 3b.

Next is the nape muscle group, which includes the upper neck muscles and the long nape muscles. For the upper neck muscles, the connection points are at the back catapophysis of the two adjacent skeletons. However, the effective span of the long nape muscles is very large, from C2 to C14. The long nape muscles connect the pleurapophysis to the back catapophysis of the two skeletons with the force lines, as shown by the green lines in Fig. 3c. Due to the effect and connection of the nape muscle group, as shown in Fig. 3c, the corresponding bio-syncretic components in the bionic structure can be presented as the bio-syncretic components in Fig. 3d.

Finally, we consider the neck abdominal muscle group, including the interspinalis muscles and the anterior cervical ligaments, as shown in Fig. 3e. The interspinalis muscle has the same effect as that discussed in the analysis above, which is also described as the vertical bio-syncretic components, as shown by the orange lines with the vertical force lines. The anterior cervical ligaments connect the front catapophysis and the pleurapophysis from C2 to C13, and thus, the effect is presented as the suspension bio-syncretic components, as shown by the green lines in Fig. 3f with the slant force lines.

According to the anatomic analysis, the connections of the rigid units in the multilevel bionic structure are determined, and it can be seen that the bio-syncretic components in each level structure are assembled symmetrically.

### 2.2 Deformation energy and external potential energy

**Fig. 4:**
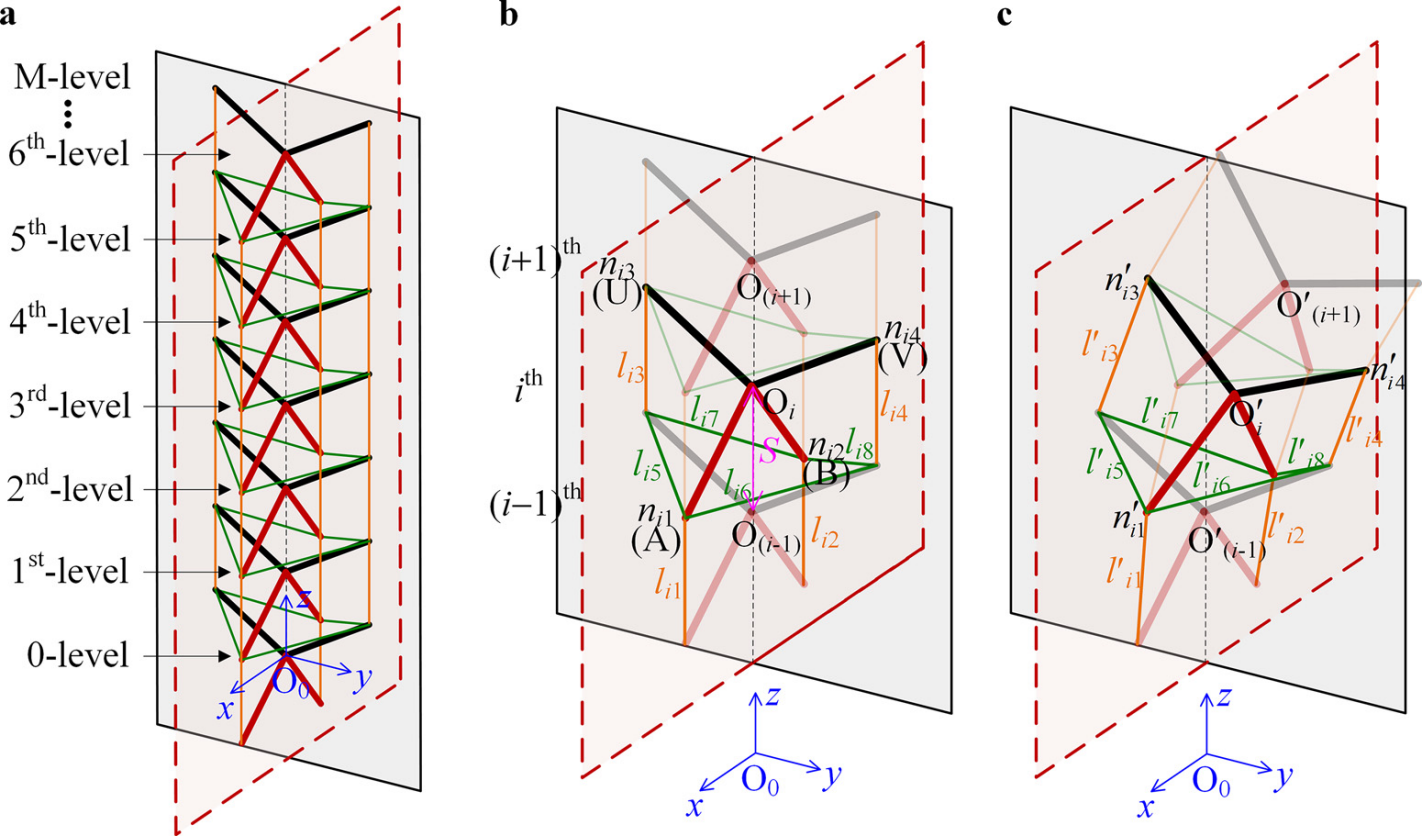
**The multilevel bionic structure and deformations.** (a) The configuration of the multilevel bionic structure; (b) configuration and assembly of the bio-syncretic components in a one-level structure with two rigid units; (c) displacements of the rigid unit and the deformations of one group of bio-syncretic components. The distance between the adjacent rigid units is *S*. The center of the rigid unit is O, and the four vertexes A, B, U, and V correspond to the front catapophysis, rear catapophysis, and two lateral catapophyses in one skeleton, labeled as *n*_1_, *n*_2_, *n*_3_, and *n*_4_. In the *i*th level structure, the eight bio-syncretic components are *l_i_*_1_, *l_i_*_2_, …, *l_i_*_8_.

After the determination of the configuration of the proposed bionic structure, the model of the multilevel bionic structure is established. In fact, the resilience characteristic for the combined deformations is the most important feature of the structure. Based on the analysis of the construction of rigid units and bio-syncretic components in the above section, the multilevel bionic structure is assembled, as shown in Fig. 4a.

Since the rigid units simulate the skeletons and the bio-syncretic components simulate the muscles, the multilevel bionic structure is constructed as shown in Fig. 4a. The one-level structure contains one rigid unit and one group of bio-syncretic components (eight components in one level), as shown in Fig. 4b. In order to unify the marks for the multilevel structure, for the *i*^th^ rigid unit as shown in Fig. 4b, the coordinate of the center is $O_{i}\in R^{1\times 3}$ (*i* = 0,1, …, M), and the coordinates of the four vertexes are expressed as $n_{ij}\in R^{1\times 3}$(*i* = 0,1, …, M, and *j* = 1, 2, 3, 4). For the *i*^th^ rigid unit, the coordinate of the vertexes is $N_{i}={\left\{n_{i1},n_{i2},n_{i3},n_{i4}\right\}}^{T}\in R^{4\times 3}$.

Before presenting the coordinates of the centers and vertexes for the displacements, we define the connection relation between the adjacent levels with a connectivity matrix **C**. Referring to Refs. [22], [23], [24], for the *i*th level, the connectivity matrix $C_{i}\in R^{8\times 8}$ (*i* = 1, 2, …, M) is introduced to describe the connection of the eight bio-syncretic components. In each connectivity matrix **C***_i_* the row vector represents the *k*th bio-syncretic component, where *k* = 1, 2, …, 8, and the *p*th column vector represents the eight vertexes *n_p_* (*p* = 1, 2, …, 8) of the (*i*−1)^th^- and *i*^th^-level rigid units. The element *c*_(_*_k_*_,_ *_p_*_)_ in **C***_i_* is equal to 1 or −1 when the bio-syncretic component connects the vertexes *n*_(_*_i_*_-1)_*_j_* or *n_ij_*. For the eight bio-syncretic components, since *p* ranges from 1 to 8, they are connected at the vertexes from *n*_(_*_i_*_-1)1_, *n*_(_*_i_*_-1),_ *n*_(_*_i_*_-1)3_, *n*_(_*_i_*_-1)4_, *n*_(_*_i_*_-1)3_, *n*_(_*_i_*_-1)4_, *n*_(_*_i_*_-1)3_, and *n*_(_*_i_*_-1)4_ to *n_i_*_1_, *n_i_*_2_, *n_i_*_3_, *n_i_*_4_, *n_i_*_1_, *n_i_*_1_, *n_i_*_2_, and *n_i_*_2_, respectively. Thus, at the *k*^th^ row element, for *n*_(_*_i_*_-1)1_, *n*_(_*_i_*_-1),_ *n*_(_*_i_*_-1)3_, *n*_(_*_i_*_-1)4_, *n*_(_*_i_*_-1)3_, *n*_(_*_i_*_-1)4_, *n*_(_*_i_*_-1)3_, and *n*_(_*_i_*_-1)4_, the element *c*_(_*_k_*_,_ *_p_*_)_ is equal to 1, and for *n_i_*_1_, *n_i_*_2_, *n_i_*_3_, *n_i_*_4_, *n_i_*_1_, *n_i_*_1_, *n_i_*_2_, and *n_i_*_2_, at the *k*^th^ row, the element *c*_(_*_k_*_,_ *_p_*_)_ is equal to −1. Each connectivity matrix **C***_i_* can be expressed as follows:

(1) $$C_{i}=\begin{array}{cc} \overset{n_{\left(i-1\right)j}}{\bar{[\begin{array}{cccc} 1 & 0 & 0 & 0\\ 0 & 1 & 0 & 0\\ 0 & 0 & 1 & 0\\ 0 & 0 & 0 & 1\\ 0 & 0 & 1 & 0\\ 0 & 0 & 0 & 1\\ 0 & 0 & 1 & 0\\ 0 & 0 & 0 & 1 \end{array}}} & \overset{n_{ij}}{\bar{\begin{array}{cccc} -1 & 0 & 0 & 0\\ 0 & -1 & 0 & 0\\ 0 & 0 & -1 & 0\\ 0 & 0 & 0 & -1\\ -1 & 0 & 0 & 0\\ -1 & 0 & 0 & 0\\ 0 & -1 & 0 & 0\\ 0 & -1 & 0 & 0 \end{array}]}} \end{array}\left(i=1,2,\ldots ,M\right)$$

Then, for the original state, as shown in Fig. 4b, the vectors of the bio-syncretic components in the *i*^th^-level structure are defined as $LC_{i}=C_{i}\cdot \left[\begin{array}{c} N_{(i-1)j}\\ N_{ij} \end{array}\right]\in R^{8\times 3}$ (*i* = 1, 2, …, M). The original lengths of the bio-syncretic components connecting the (*i*−1)^th^-level and *i*th-level rigid units are defined as $L_{i}={\left\{l_{i1},l_{i2},\ldots ,l_{i8}\right\}}^{T}\in R^{8\times 1}$ (*i* = 1, 2, …, M), given by

(2) $$\begin{array}{c} L_{i}=∥LC_{i}∥\in R^{8\times 1}\left(i=1,2,\cdots ,M\right) \end{array}$$

For instance, at the *i*th level, the original lengths of the eight bio-syncretic components are given by

(3) $$\begin{array}{c} l_{i1}=∥n_{\left(i-1\right)1}-n_{i1}∥,l_{i2}=∥n_{\left(i-1\right)2}-n_{i2}∥,l_{i3}=∥n_{\left(i-1\right)3}-n_{i3}∥,l_{i4}=∥n_{\left(i-1\right)4}-n_{i4}∥\\ l_{i5}=∥n_{\left(i-1\right)3}-n_{i1}∥,l_{i6}=∥n_{\left(i-1\right)4}-n_{i1}∥,l_{i7}=∥n_{\left(i-1\right)3}-n_{i2}∥,l_{i8}=∥n_{\left(i-1\right)4}-n_{i2}∥ \end{array}$$

which conforms to Fig. 4b.

With the action of an external force system, the multilevel bionic structure is deformed, and six-direction combined deformation of each rigid unit occurs, as shown in Fig. 4c. For each rigid unit, we define the translation displacement matrix as $D_{i}={\left\{x_{i},y_{i},z_{i}\right\}}^{T}$ and the rotation displacement matrix as $\phi_{i}={\left\{\phi_{ix},\phi_{iy},\phi_{iz}\right\}}^{T}$, and thus, the displacement of the *i*^th^ rigid unit is defined as $d_{i}=D_{i}⊕\phi_{i}={\left\{x_{i},y_{i},z_{i},\phi_{ix},\phi_{iy},\phi_{iz}\right\}}^{T}\in R^{6\times 1}$. The rotation transformation matrix **Φ***_i_* is given by

$$\Phi_{i}=\left[\;\begin{array}{ccc} \cos\phi_{iz}\cos\phi_{iy}\;\;\;\; & \;\;\;\;\cos\phi_{iz}\sin\phi_{iy}\sin\phi_{ix}-\sin\phi_{iz}\cos\phi_{ix}\;\;\;\; & \;\;\;\;\cos\phi_{iz}\sin\phi_{iy}\cos\phi_{ix}+\sin\phi_{iz}\sin\phi_{ix}\\ \sin\phi_{iz}\cos\phi_{iy}\;\;\;\; & \;\;\;\;\sin\phi_{iz}\sin\phi_{iy}\sin\phi_{ix}+\cos\phi_{iz}\cos\phi_{ix}\;\;\;\; & \;\;\;\;\cos\phi_{iz}\sin\phi_{iy}\cos\phi_{ix}+\sin\phi_{iz}\sin\phi_{ix}\\ -\sin\phi_{iy}\;\;\;\; & \;\;\;\;\cos\phi_{iy}\sin\phi_{ix}\;\;\;\; & \;\;\;\;\cos\phi_{iy}\cos\phi_{ix} \end{array}\;\right]$$

For the six-direction displacements of each rigid unit, the coordinate of the center is ${O^{\prime }}_{i}\in R^{1\times 3}$ (*i* = 0,1, …, M), which depends on the translational displacements **D***_i_*. The coordinates of the four vertexes are ${n^{\prime }}_{ij}\in R^{1\times 3}$ (*i* = 0,1, …, M; *j* = 1, 2, 3, 4), which depend on both the translational displacement matrix **D***_i_* and the rotational displacement matrix **ϕ***_i_*. The coordinates of the four vertexes of the rigid unit at the *i*th level can be written as ${N^{\prime }}_{i}={\left\{{n^{\prime }}_{i1},{n^{\prime }}_{i2},{n^{\prime }}_{i3},{n^{\prime }}_{i4}\right\}}^{T}\in R^{4\times 3}$. Then, the relationship of the vertexes between **n***_ij_* and ${n^{\prime }}_{ij}$ can be written as

(4) $$\begin{array}{c} {{n^{\prime }}_{\mathrm{ij}}}^{T}=\Phi_{i}\cdot {n_{\mathrm{ij}}}^{T}+D_{i}\left(i=0,1,\cdots ,M,j=1,2,3,4\right) \end{array}$$

The deformed lengths of the eight bio-syncretic components connecting the (*i*−1)^th^-level and the *i*th-level rigid units are defined as ${L^{\prime }}_{i}={\left\{{l^{\prime }}_{i1},{l^{\prime }}_{i2},\ldots ,{l^{\prime }}_{i8}\right\}}^{T}\in R^{8\times 1}$ (*i* = 1, 2, …, M), which can be calculated with the following equation:

(5) $$\begin{array}{c} {L^{\prime }}_{i}=∥C_{i}\cdot \left[\begin{array}{c} {N^{\prime }}_{\left(i-1\right)j}\\ {N^{\prime }}_{\mathrm{ij}} \end{array}\right]∥\in R^{8\times 1}\left(i=1,2,\cdots ,M\right) \end{array}$$

For the *i*^th^-level structure, the deformed lengths of one group of bio-syncretic components are written similarly as Eq. 3. For instance, the first bio-syncretic components in one group are written as $l_{i1}^{\prime }=∥{n^{\prime }}_{(i-1)1}-{n^{\prime }}_{i1}∥$, where each ${n^{\prime }}_{ij}$ is defined as in Eq. 4.

In summary, for the M-level bionic structure, there are M+1 rigid units and M groups of bio-syncretic components. For the M+1 rigid units, there are M+1 centers and 4(M+1) vertexes. The coordinates of the centers are written as $O={\left\{O_{0},O_{1},O_{2},\ldots ,O_{M}\right\}}^{T}\in R^{(M+1)\times 3}$, the coordinates of the vertexes are written as $N={\left\{N_{0},N_{1},N_{2},\ldots ,N_{M}\right\}}^{T}\in R^{4(M+1)\times 3}$, and the original lengths of the M group bio-syncretic components are $L={\left\{L_{1}^{T},L_{2}^{T},\ldots ,L_{M}^{T}\right\}}^{T}\in R^{8M\times 1}$. For the deformations, for the displacements of each rigid unit **d***_i_*, the coordinates of centers are ${O^{\prime }}_{i}=d_{i}+O_{0}O_{i}=\left\{x_{i},y_{i},iS+z_{i}\right\}$, where $O^{\prime }={\left\{{O^{\prime }}_{0},{O^{\prime }}_{1},{O^{\prime }}_{2},\ldots ,{O^{\prime }}_{M}\right\}}^{T}\in R^{(M+1)\times 3}$. The coordinates of the vertexes after deformation are $N^{\prime }={\left\{{N^{\prime }}_{0},{N^{\prime }}_{1},{N^{\prime }}_{2},\ldots ,{N^{\prime }}_{M}\right\}}^{T}\in R^{4(M+1)\times 3}$, and the deformed lengths of the bio-syncretic components are $L^{\prime }={\left\{{{L^{\prime }}_{1}}^{T},{{L^{\prime }}_{2}}^{T},\ldots ,{{L^{\prime }}_{M}}^{T}\right\}}^{T}\in R^{8M\times 1}$. Since the 0-level rigid unit is fixed on the ground, its center coordinate is $O_{0}={O^{\prime }}_{0}={\left\{0,0,0\right\}}^{T}$, and the values of the vertex coordinates of **N**_0_ and ${N^{\prime }}_{0}$ are constant.

For the M-level bionic structure, the potential energy is associated with the deformation of the bio-syncretic components. The physics constitutive function of each bio-syncretic component *F_ik_* is assumed to be

(6) $$\begin{array}{c} F_{\mathrm{ik}}=f_{\mathrm{ik}}\left(\xi_{\mathrm{ij}}\right)\left(i=1,2,\cdots ,M,k=1,2,\cdots ,8\right) \end{array}$$

where *ξ_ij_* is the local axial coordinate of each bio-syncretic component. The physics constitutive functions *f_ik_* represent the nonlinear relationship between the force and deformation of each bio-syncretic component, as shown in Fig. 4. The deformation potential of the bio-syncretic components is defined as *V_e_*, given by

(7) $$\begin{array}{c} V_{e}=\sum_{i=1}^{M}\sum_{k=1}^{8}\int_{l_{\mathrm{ik}}}^{{l^{{}^{\prime }}}_{\mathrm{ik}}}f\left(\xi_{\mathrm{ik}}\right)d\xi_{\mathrm{ik}} \end{array}$$

where *l_ik_* and $l_{ik}^{\prime }$ are the lengths for the original state and the deformed state of each bio-syncretic component in the *i*^th^-level structure, as defined in Eqs. 2 and 5, respectively.

However, for the M-level structure with M+1 rigid units, as shown in Fig. 4a, since the rigid part for the 0-level is fixed on the ground, there are M rigid units in the displacements. The potential energy associated with the masses of the rigid units in the system is defined as *V_g_*, given by

(8) $$\begin{array}{c} V_{g}=\sum_{i=1}^{M}m_{i}gz_{i} \end{array}$$

where *z_i_* is the displacement of each rigid unit in the *z*-direction.

According to Eqs. 7, 8, the potential energy of the multilevel bionic structure is obtained, based on which the mechanical model of the bionic structure can be obtained.

For an external force system, defined as $A=\left\{A_{i}\right\}\in R^{M\times 6}$, where $A_{i}=F_{i}⊕M_{i}\in R^{1\times 6}$, $F_{i}=\left\{F_{ix},F_{iy},F_{iz}\right\}\in R^{1\times 3}$, and $M_{i}=\left\{M_{ix},M_{iy},M_{iz}\right\}\in R^{1\times 3}$, the external energy *E_ex_* is given by

(9) $$\begin{array}{c} E_{\mathrm{ex}}=\sum_{i=1}^{M}\left\{F_{i}\cdot D_{i}+\left[\left(P_{i}\times F_{i}\right)+M_{i}\right]\cdot \phi_{i}\right\} \end{array}$$

where $P_{i}\in R^{1\times 3}$ represents the vector between the center of each rigid unit and the action point of **F***_i_*. The total potential energy of the M-level bionic structure is defined as

(10) $$\begin{array}{c} \Pi =V-E_{\mathrm{ex}} \end{array}$$

According to the principle of the minimum potential energy, for the accurate integral form with the action of an external force system, the total energy of the system should be at a minimum. The following section establishes the integral form-finding method for illustrating the flexible combination deformations with different force systems.

## 3 Deformations and integral form-finding

For the bionic M-level structure, there are M groups of bio-syncretic components and M movable rigid units. The potential energy *V* of the M-level structure containing the deformation potential energy and the gravitational potential energy is written as *V* = *V_e_* + *V_g_*, where *V_e_* and *V_g_* are defined as shown in Eqs. 7 and 8, respectively.

Based on the principle of minimum potential energy, the displacements of each rigid unit satisfy the following condition

(11) $$\begin{array}{c} \delta \left[\Pi \right]=0\Leftrightarrow L\left[V-E_{\mathrm{ex}}\right]=0 \end{array}$$

where the operator **L**[⋅] represents the partial derivative to the generalized coordinates as ${\left\{\frac{\partial }{\partial x_{i}},\frac{\partial }{\partial y_{i}},\frac{\partial }{\partial z_{i}},\frac{\partial }{\partial \phi_{ix}},\frac{\partial }{\partial \phi_{iy}},\frac{\partial }{\partial \phi_{iz}}\right\}}^{T}$

Therefore, utilizing the principle of minimum potential energy in Eq. 10, it represents a group of nonlinear algebraic equations with 6 × M equations with respect to the 6 × M displacements. Solving the 6 × M algebraic equations in Eq. 11, the displacements of each rigid unit and the integral form can be obtained with the Newton-Raphson method.

In the following illustration, two cases for the integral form-finding process are shown. The first case is the integral form for a structure slant. When the structure is in a slant, the weight of each rigid unit induces transverse bending deformation, which simulates the deformation features of the physiological scoliosis phenomenon for the neck. The second case is the bionic structure with torsion and axial combined deformations.

### 3.1 Slant

**Fig. 5:**
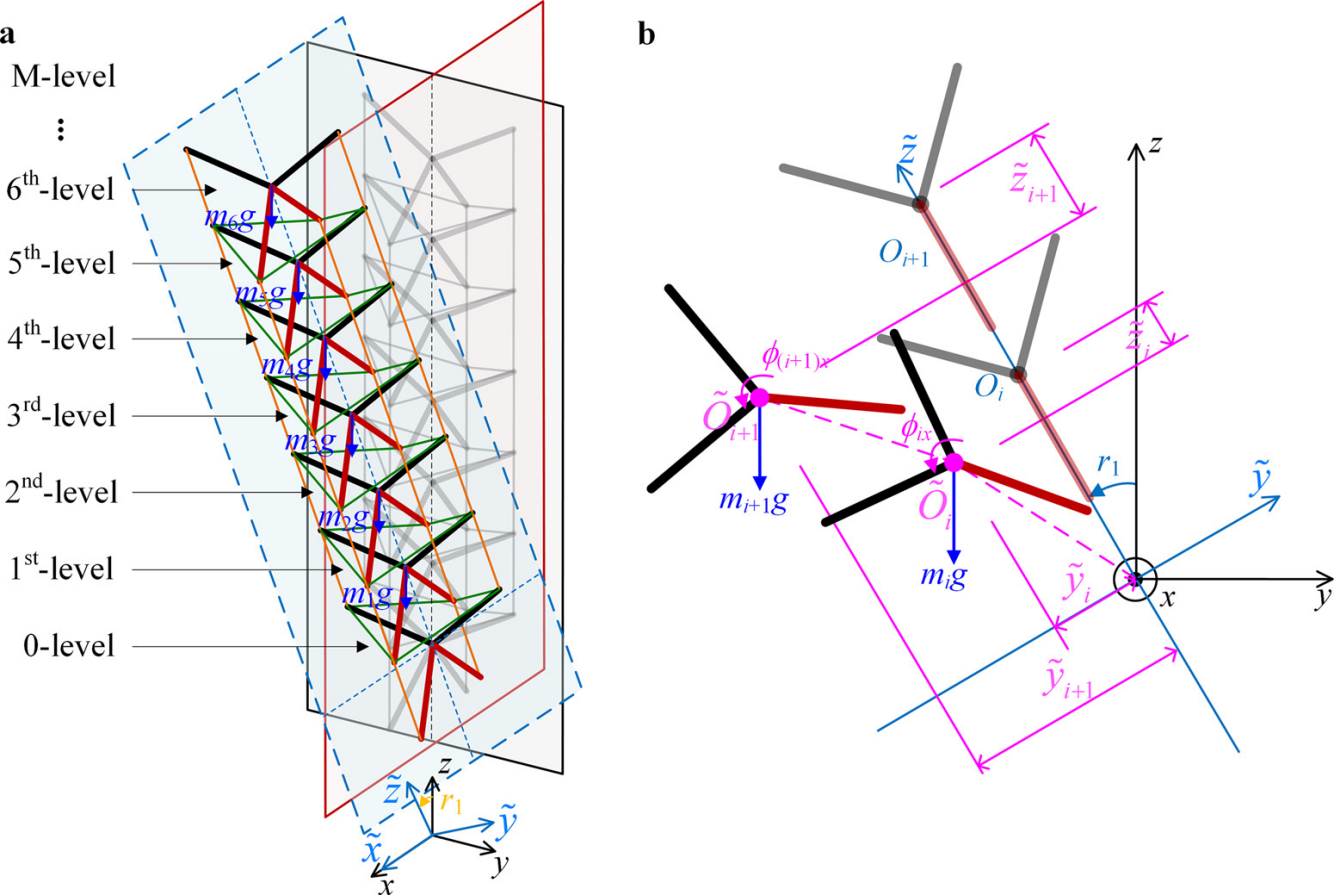
**The multilevel bionic structure in slant.** (a) M-level structure for the slant angle *r_x_* around the *x* axis. (b) Displacements occurring in the plane $\left(\tilde{y},\tilde{z}\right)$ with gravity. For the slant angle *r_x_* around the *x*-axis, the potential energy is established in the coordinate system $\left(x,\;\tilde{y},\;\tilde{z}\right)$.

In this case, the flexible large deformation illustrates the accuracy of the mechanical model of the bionic structure. Assuming that the bionic structure is in an original slant angle occurring around the *x*-axis (imitation of a tilting head) as *r_x_*, the coordinate axis contains $\tilde{y}$ and$\;\tilde{z}$, as shown in Fig. 5a. The axial direction of the bionic structure is along the coordinate axis $\tilde{z}$, and the transverse bending deformation occurs in the plane ($\tilde{y},\;\tilde{z}$), as shown in Fig. 5b.

The deformation potential energy of the slant case is written as ${\tilde{V}}_{e}$ and has the same function relationship with the displacements as that for Eq. 6, which is given by

(12) $$\begin{array}{c} {\tilde{V}}_{e}=\sum_{i=1}^{M}\sum_{k=1}^{8}\int_{{\tilde{l}}_{\mathrm{ik}}}^{{\tilde{l^{\prime }}}_{\mathrm{ik}}}f\left(\xi_{\mathrm{ik}}\right)d\xi_{\mathrm{ik}} \end{array}$$

where $\tilde{l}{}_{ik}^{\prime }$ and ${\tilde{l}}_{ik}$ represent the deformed lengths and original lengths of the M groups of bio-syncretic components in the coordinate system $\left(x,\;\tilde{y},\;\tilde{z}\right)$. The gravity of each rigid unit is considered to induce the external force potential energy, defined as ${\tilde{E}}_{ex}$ and written as

(13) $$\begin{array}{c} {\tilde{E}}_{\mathrm{ex}}=\sum_{i=1}^{M}m_{i}g\left\{-\text{sin}r_{x},\;-\text{cos}r_{x}\right\}\left\{\begin{array}{c} {\tilde{y}}_{i}\\ {\tilde{z}}_{i} \end{array}\right\} \end{array}$$

The principle of the minimum potential energy in the new coordinate system of $\left(x,\;\tilde{y},\;\tilde{z}\right)$ is written as

(14) $$\delta \left[\tilde{\prod }\right]=\delta \left[{\tilde{V}}_{e}-{\tilde{E}}_{ex}\right]=0$$

Based on the principle of minimum potential energy, as shown in Eq. 14, the integral forms of the structure are determined for different values of the slant angle *r_x_* induced by gravity.

In this study case, to clearly show the effect of the external action on the integral form of the structure, which is also influenced by the original slant angle and the structural parameters, and to illustrate the effect of the structural configuration on the nonlinear mechanical properties rather than the nonlinear material constitutive of the bio-syncretic component, the constitutive function of each bio-syncretic component is assumed to be a linear function with the linear stiffness coefficient *k_ik_*, written as

(15) $$\begin{array}{c} f_{\mathrm{ik}}\left(\xi_{\mathrm{ik}}\right)=k_{\mathrm{ik}}\xi_{\mathrm{ik}} \end{array}$$

Then, the corresponding deformation potential energy ${\tilde{V}}_{e}$ is written as

(16) $$\begin{array}{c} {\tilde{V}}_{e}=\sum_{i=1}^{M}\sum_{k=1}^{8}\frac{1}{2}k_{\mathrm{ik}}{\left({\tilde{l^{\prime }}}_{\mathrm{ik}}-{\tilde{l}}_{\mathrm{ik}}\right)}^{2} \end{array}$$

**Fig. 6:**
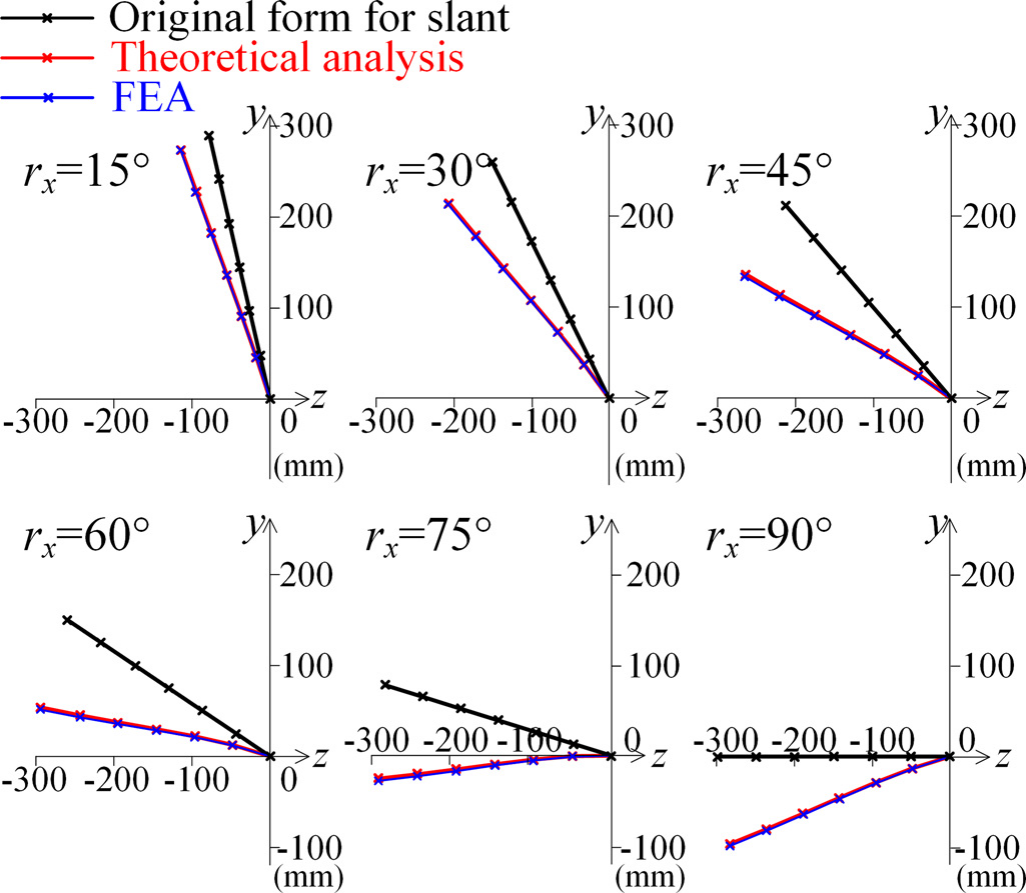
**Comparison of the integral form of the structure in the plane (*y, z*) with gravity for the theoretical analysis and the Finite Element Analysis (FEA) for different values of the slant angle *r_x_*.** The crosses represent the centers of the rigid units.

**Fig. 7:**
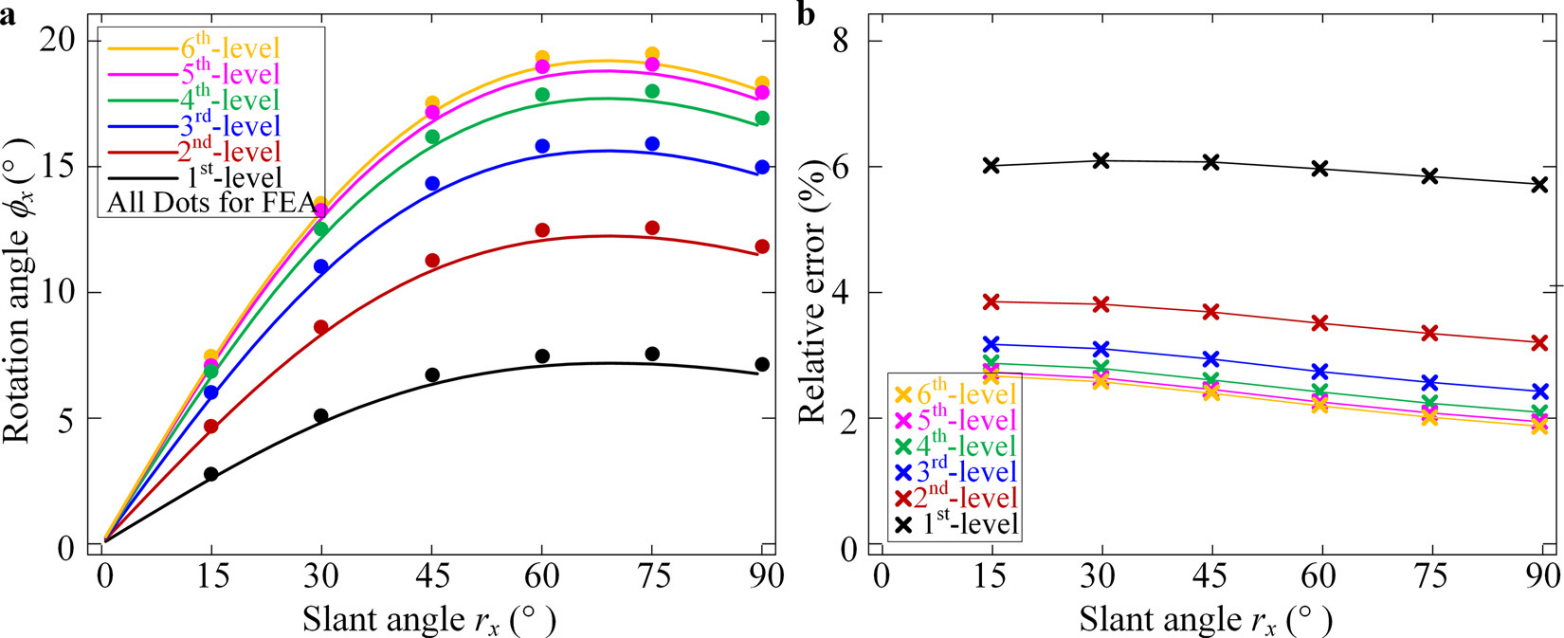
**The rotation displacements obtained by theoretical analysis and FEA.** (a) Comparisons of the rotation angle *ϕ_x_* for the theoretical analysis (Lines) and the FEA (Dots) of each level of the rigid unit for different values of the slant angle *r_x_*. (b) Relative error for the theoretical analysis and the FEA for each level of the rigid unit for different values of the slant angle *r_x_*.

**Fig. A1:**
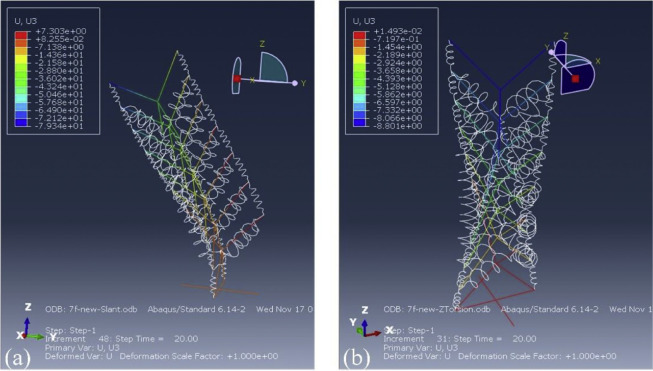
**The model for the bionic structure in the ABAQUS for (a) the slant case for slant angle as*****r*****=45° under gravity, and (b) torsion with*****T**_**i**_***.**

According to the principle of minimum potential energy, as shown in Eq. 13, for the slant angle *r_x_*, the integral form can be obtained. For the FEA, the software ABAQUS and the modeling process are shown in Fig. A1 in the Appendix. The locations of each rigid unit in the structure in the original coordinate plane (*y, z*) obtained with the theoretical analysis and the FEA are shown in Fig. 6. In addition, the rotation displacements *ϕ_x_* of each rigid unit for the theoretical analysis and the FEA are compared in Fig. 7. Because the distance between the two skeletons is on the order of a centimeter, such as the woodpecker in Refs. [35,[38], [39], [40]], the distance is fixed as *S* = 50 mm in the study case. Additionally, the other structural parameters are fixed as *R_a_* = *R_b_* = *R_u_* = *R_v_* = 100 mm and *θ_a_* = *θ_b_* = *θ_u_* = *θ_v_* = 45°. Furthermore, because one muscle fiber has a stiffness of 0.01–0.02 N⋅m^−1^ and one bunch of muscle has thousands of muscle fibers, the stiffness of one bio-syncretic component is assumed to be *k_ik_* = 10 N⋅m^−1^. All the values and errors of the displacements obtained with the theoretical analysis and the FEA are listed in Table 1 in the Appendix.

**Table 1:** **The comparison of displacements between theorical analysis and FEA.** (Blue background box for the theorical analysis, Orange background box for FEA, and Gray background box for the Error).

For the comparisons of the plane transverse bending displacements occurring in the plane (*y, z*) between the theoretical results from Eq. 14 and the FEA, as shown in Figs. 6 and 7, the integral form-finding method can be applied for large flexible combined deformations, and the integral form has high accuracy. In addition, the displacements obtained with the FEA are slightly larger than the results obtained with the theoretical model, since the rods are elastic rather than a rigid unit in the finite element (FE) model.

For the variation of the rotation displacements *ϕ_x_* for each level of the rigid unit shown in Fig. 7, the relationship between the rotation angle *ϕ_x_* and the slant angle *r_x_* is nonlinear. For the increase of the slant angle *r_x_*, the displacement *ϕ_x_* increases and then slightly decreases for the slant angle beyond about 70°. For the increase of the slant angle, the weight of each rigid unit induces a larger bending deformation, while the slant angle further increases, as shown in Fig. 6 (for *r_x_* = 75° and *r_x_* = 90°), and when the integral form of the structure is under the *y*-axis, the four bio-syncretic components in the axial direction are more involved in the resistance to deformations.

### 3.2 Torsion and compression combination deformations

The proposed multilevel bionic structure can flexibly deform in torsion, which is the most significant feature of a deformable structure. The following analysis discusses the variation of the displacements and deformations during torsional action, which guide the design of structure for further study on dynamic behaviors, stability, and control in torsion. In this case, the structural parameters are fixed to be the same as those for the above case.

**Fig. 8:**
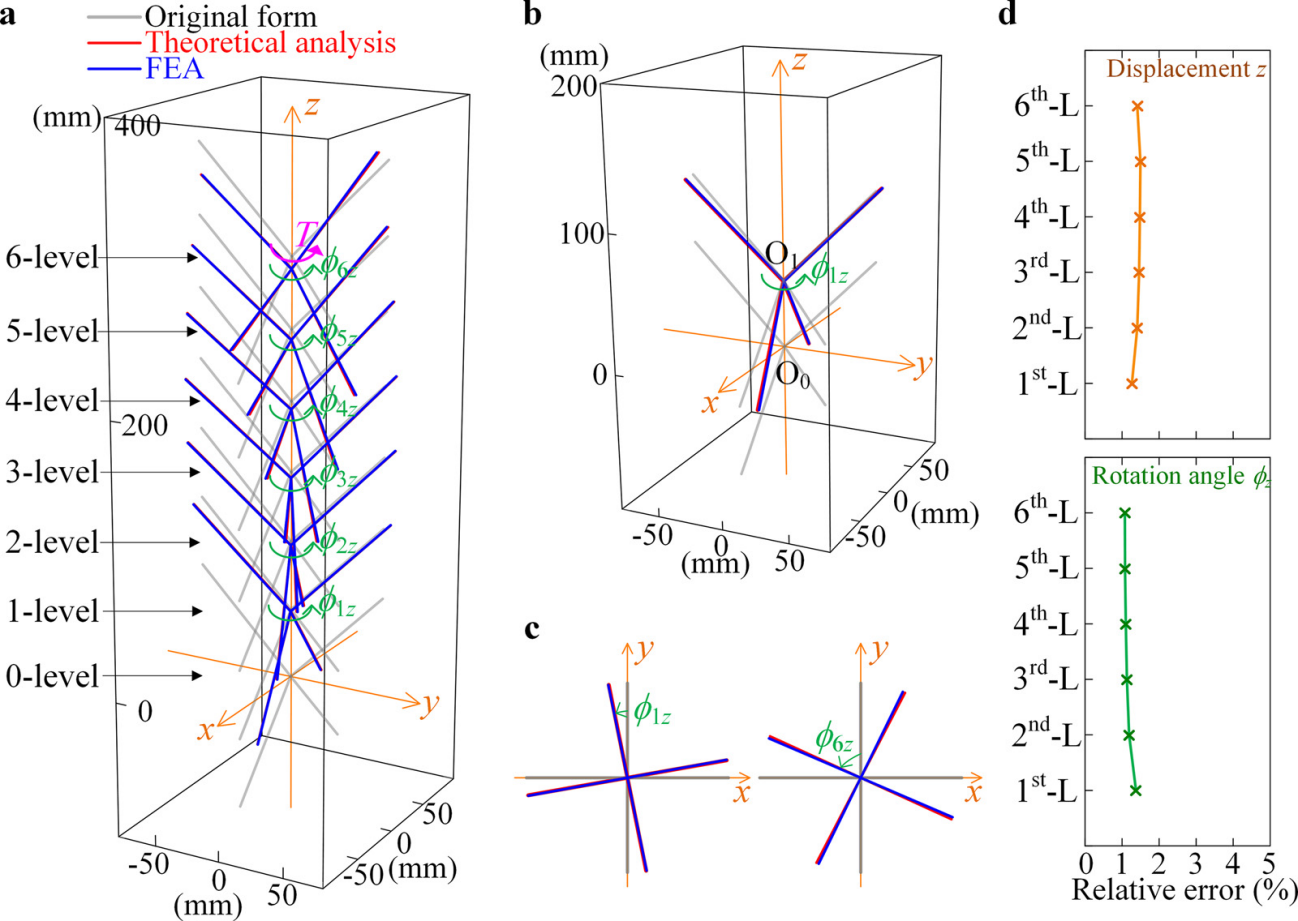
**The torsion displacement *ϕ_iz_* and the axial displacement *z_i_* by the theoretical analysis and the FEA.** (a) Comparison of integral form between the theoretical analysis and the FEA for torsion applied on the upper level. (b) Comparison of displacement of the first-level rigid unit between the theoretical analysis and the FEA. (c) Rotation displacement of the first-level rigid unit *ϕ*_1_*_z_* and the sixth-level rigid unit *ϕ*_6_*_z_* for the projection on the plane (*x, y*). (d) Relative error between the theoretical analysis and the FEA for the displacement *z* and the torsion rotation angle *ϕ_z_*.

Similar to the previous case study, considering a seven-level bionic structure, we apply a torsion *T* to the top rigid unit. Therefore, in the matrix of the externally acting spatial arbitrary force system **A**, only *A*_66_ is a non-zero element, as *A*_66_ = *M*_6_*_z_* = *T*. Then, according to the principle of minimum potential energy defined by Eq. 11, the integral form and the displacements are obtained. Fig. 8 illustrates the displacements of each level of the rigid unit and the comparison between the theoretical results for Eq. 11 and the FEA. The values and errors of *ϕ_iz_* obtained with the theoretical analysis and the FEA are listed in Table 2 in the Appendix.

**Table 2:** **The displacement*****ϕ**_**z**_***for torque*****T**_**i**_***=10 N⋅mm active on the top level.** (Blue background box for the theorical analysis, Orange background box for FEA, and Gray background box for the Error).

For the torsional action, the structure is in a large combination deformation state with both torsional and axial deformations. In addition, for the comparison of the integral form between the theoretical analysis and the FEA, the modeling and integral form-finding method accurately display the combination deformations of the structure. As shown in Fig. 8c, the small error of *ϕ*_6_*_z_* between the theoretical analysis and the FEA comes from the elasticity of rods in the FEA. In the theoretical analysis, the rods, reflecting the skeleton section, are considered to be a rigid body, while in the FEA, the rods can have tiny deformations. Although the elastic deformations of the rods in the FEA are much smaller compared to those in the bio-syncretic components, the elastic deformations of the rods induce the relative error of the results between the theoretical analysis and the FEA. As shown in Fig. 8d, the relative error increases only slightly for the torsion rotation angle *ϕ_z_* for the lower level since the equivalent force acting on the lower-level structure is larger. However, there is little difference in the axial displacement since the weight of each skeleton is much smaller than the head.

Based on the above analysis, the constitution of the bird neck provides inspiration for a multilevel flexible bionic structure that has potential application in different fields. Additionally, the mechanical properties of the bionic structure can explain the biomechanical phenomenon and some functions of the bird neck.

## 4 Explanation for biomechanical phenomenon

### 4.1 Natural form of gravity

**Fig. 9:**
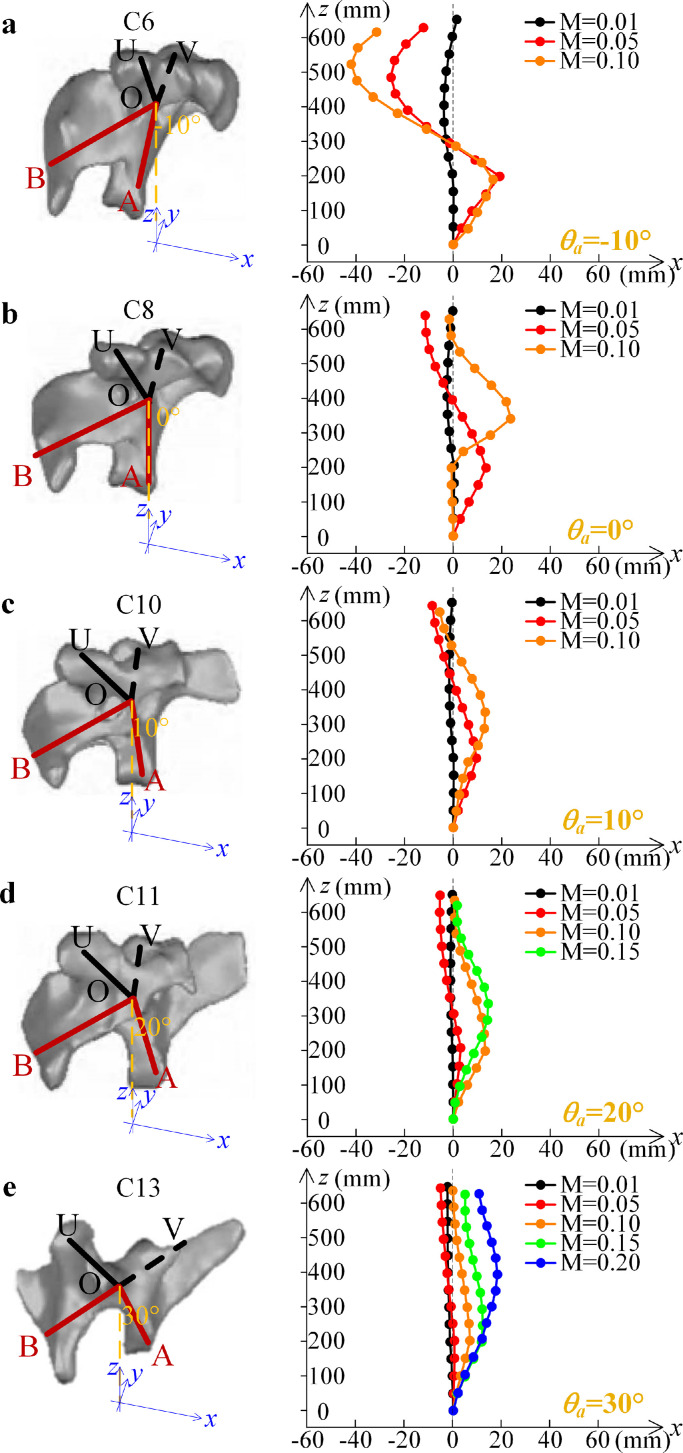
**Forms of the bionic structure with different masses of the end effector and the positions of each rigid unit.** (a) *θ_a_* = −10° (−*π*/18), (b) *θ_a_* = 0°, (c) *θ_a_* = 10° (*π*/18), (d) *θ_a_* = 20° (*π*/9), and (e) *θ_a_* = 30° (*π*/6). (Dots for the centers of the rigid units). The configuration of each skeleton is referenced from Refs. [34], [35].

In the above case studies for the verification of the modeling, each rigid unit in the bionic structure is assumed to have a fully symmetric tortile “X” shape, and the relevant structural parameters are assumed to be *R_a_* = *R_b_* = *R_u_* = *R_v_* and *θ_a_* = *θ_b_* = *θ_u_* = *θ_v_*. However, in the real bird neck structure, as shown in Figs. 1 and 2, the distances of the catapophysis to the center of this skeleton are different. As shown in the anatomic analysis in Fig. 1, for the skeletons, in the neck structure, the most obviously different parameter is the angle of the front catapophysis (Line OA of the rigid unit in the bionic structure). From the top of the skeleton to the bottom, the upper two sides of the catapophysis are symmetrical as *R_u_* = *R_v_* and *θ_u_* = *θ_v_*, while the front and rear catapophyses are asymmetrical. Since the asymmetry occurs in the (*x, z*) plane, for the gravitational loading, the deformation of the bionic structure occurs in the (*x, z*) plane. Assuming that the mass of the end effector (the head) is *M* and that of each rigid unit (the skeleton) is *m_i_*, the form of the bionic structure should satisfy the following optimum condition:

(17) $$\begin{array}{c} \text{Min}∥V_{e}\left(x_{i},z_{i},\phi_{\mathrm{iy}}\right)∥\\ s.t.\frac{\partial V_{e}}{\partial z_{i}}=\sum_{i}^{N}m_{i}g+\mathrm{Mg} \end{array}$$

where *V_e_* is the deformation potential energy, defined using the same expression as in Eq. 7. Based on the optimum condition, Eq. 17, the bionic structure can provide sufficient loading capacity for the upper gravity in the axial direction, and the displacements (*x_i_, z_i_, ϕ_iy_*) of each rigid unit meet the requirements of the least deformation energy *V_e_* of the bio-syncretic components. In order to utilize the integral form obtained with Eq. 17 to explain the natural configuration of the bird neck, we consider that there are 14 rigid units in the bionic structure. Since it is known that the angle of the front catapophysis is different for different levels of the skeleton, the structural parameter *θ_a_* for different rigid units varies. For different *θ_a_*, for the increase of the mass of the head, the integral forms determined using Eq. 17 are shown in Fig. 9.

Because there are 14 rigid units in the structure and the angles of the front catapophysis are fixed as *θ_a_* = −10°, 0°, 10°, 20°, 30°, the integral forms are obtained with gravity. Based on the integral forms in Fig. 9, the forms for the type of “S” fit the natural configuration of the bird necks. Thus, the bionic structure presents a minimal energy structure with gravity. According to the integral forms shown in Fig. 9, the bending deformation occurs in the direction with lower flexural rigidity, and the axial deformation occurs for squeezing due to gravity. When the angle of the front catapophysis points toward the rear direction as *θ_a_* = −10°, the displacements for each rigid unit are much larger than those for the other cases. With the increase of the angle *θ_a_*, these integral forms are compared. Since the flexural rigidity around the *y*-axis increases, the global deformation of the structure is reduced and the loading capacity increases. The integral forms of the bionic structure can explain the natural configuration of the bird necks, as shown in Fig. 1a. In the bird necks, the angles of the front catapophysis in the lower parts of the skeletons are larger than the values in the upper parts of the skeletons since the lower levels of the skeletons and muscles need to load a larger upper mass, which verifies the autopsy results in Refs. [34,35].

The above analysis shows the static mechanical property of the bionic structure, which provides an explanation of the natural configuration of the bird necks. Then, the following analysis provides the mechanism and advantages of the natural configuration of the neck birds with dynamic properties’ analysis.

### 4.2 Dynamic stabilization for the bending motion

**Fig. 10:**
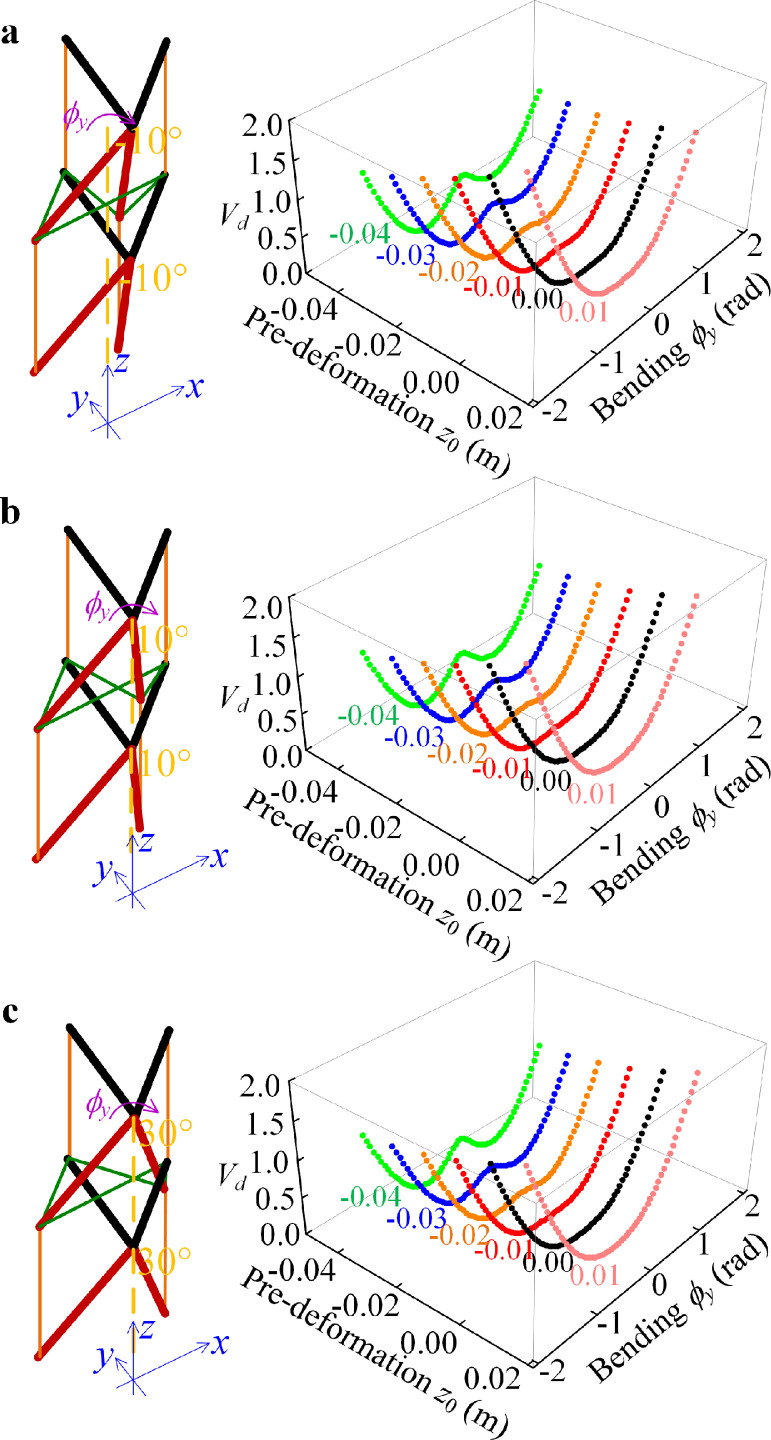
**Deformation potential energy for the plane bending around the *y*-axis for different values of the pre-deformation in the axial direction and different structural parameters.** (a) The potential energy and equilibrium for bending with different axial pre-deformations when the structural angle is as *θ_a_* = −10°, (b) The potential energy and equilibrium for bending with different axial pre-deformations when the structural angle is as *θ_a_* = 10°, and (c) The potential energy and equilibrium for bending with different axial pre-deformations when the structural angle is as *θ_a_* = 30°.

According to the above results, the natural configuration of the bird neck is in the “S” form, and we wish to determine the reason and mechanism for the dynamic stabilization of the bird neck based on the bionic structure. The bird neck structure mainly plays the role of dynamic stabilization in the bending direction for flight or walking [22], [23], [24]. For the loading of the upper mass, the deformation in the axial direction is defined as *z*_0_, and then, the variation of the potential energy for the bending deformation of the bionic structure is analyzed to illustrate the mechanism of the dynamic stabilization function of the bird neck. Two arbitrary levels of the rigid units in the bionic structure are chosen, as shown in Fig. 10. The major difference between the two levels of skeletons is the angle of the front catapophysis. In the bionic structure, the values of *θ_a_* are fixed as −10°, 10°, and 30° to simulate the different levels of the skeletons, i.e., C4-C5, C10-C11, and C13-C14.

For the deformation potential energy for bending shown in Fig. 10, it can be seen that for pre-compression in the axial direction (in the *z*-direction), the multi-steady state phenomenon occurs. Because the pre-stretching deformation *z*_0_ is a positive value, there is only one stable zero equilibrium. For pre-compression, with the increase of the magnitude of *z*_0_, the zero equilibrium becomes unstable and there are two new stable equilibriums. Therefore, the variable stiffness property for bending can be achieved with the pre-deformation in the axial direction. The variable stiffness property is in demand in vibration isolation. Since the proposed bio-inspired structure has pre-deformation in the axial direction with the upper loading, the linear stiffness coefficient in the bending direction is reduced, which can widen the effective isolation band. Thus, the proposed bionic structure provides the design concept of torsion or bending direction for vibration isolation.

**Fig. 11:**
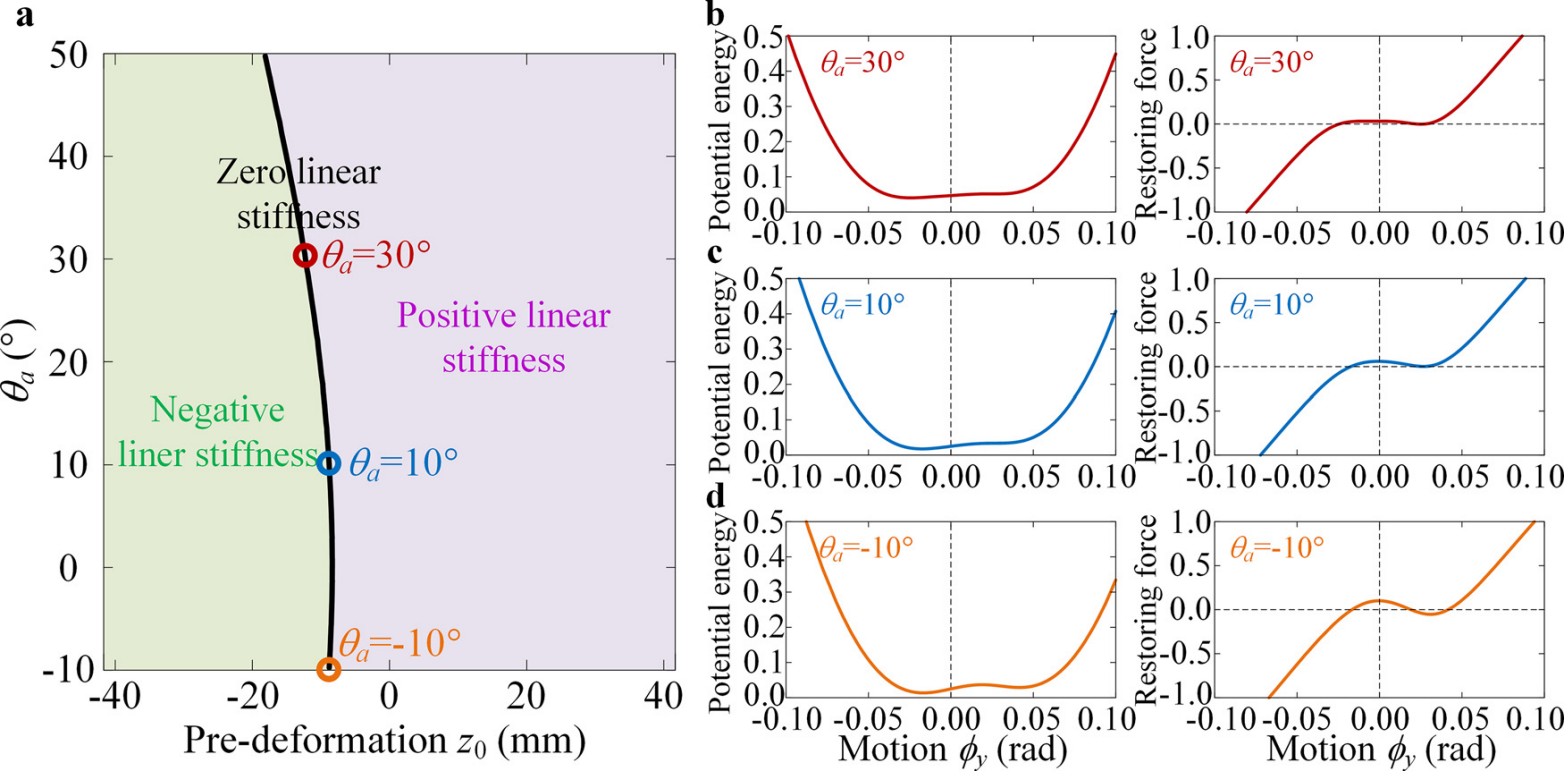
**The structural parameters regions for different number of equilibrium and the potential energies in different regions.** (a) Relationship between pre-deformation *z*_0_ in the axial direction and different structural parameters *θ_a_* for the linear dynamic stiffness equal to zero. The deformation potential energy and the restoring force for plane bending around the *y*-axis for different values with (b) *θ_a_* = −10°, (c) *θ_a_* = 10°, and (d) *θ_a_* = 30°. In the green region of the structural parameter plane, the linear stiffness coefficient is negative and the system has multiple equilibrium; in the purple region, the linear stiffness coefficient is positive and the system has only one equilibrium; when the structural parameters are fixed on the black line, the linear stiffness coefficient equals to zero and system would display the QZS property.

For the bionic structure utilized in dynamic stabilization based on the vibration isolation concept, the design principle for the quasi-zero stiffness (QZS) property can be proposed as

(18) $$\begin{array}{c} r_{\text{QZS}}=\left\{\left(\mu ,z_{0}\right)|{\frac{\partial^{2}V_{d}\left(\mu ,z_{0}\right)}{\partial \phi_{y}^{2}}|}_{\phi_{y}=0}=0\right\} \end{array}$$

where **μ** represents the structural parameters including *θ_a_*, and *z*_0_ is the pre-deformation in the axial direction. Using the design criteria of Eq. 18, the structural parameter *θ_a_* for the QZS property for different *z*_0_ is obtained, and the corresponding potential energy and the restoring force of the structure are shown in Fig. 11.

For the condition of linear dynamic stiffness equal to zero for the QZS property, the relationship between the pre-deformation in the axial direction and the structural parameter is shown in Fig. 11a. From the potential energy curves and restoring forces on the QZS condition line, the bionic structure has an equivalent zero linear dynamic stiffness as well as a positive restoring force, as shown in Fig. 11b–d. For a larger *θ_a_*, the restoring force at the zero point for bending is larger, which corresponds to the biological feature that the lower levels in the bird neck need better static stability.

In summary, for pre-deformation in the axial direction induced by gravity, each section of the bionic structure can realize a QZS dynamic property, which explains the significant bending dynamic stability of the bird neck for continuous low-frequency excitation in flight and while walking.

## 5 Conclusion

Bio-inspired by the significant orientation effect, a novel bionic bird-neck rigid-flexible structure is proposed and the integral form-finding method for flexible combination deformation is proposed. The proposed structure has high spatial accessibility. The integral form-finding method is adopted for flexible combination deformations. The following results and conclusions are obtained.

(1) The bionic mechanism of the proposed multilevel rigid-flexible structure is clearly demonstrated in this study. The shape of the rigid units and the connection of the bio-syncretic components are determined with the analysis of the construction of the bird neck structure. The bionic multilevel structure has high spatial accessibility in different directions.

(2) For combined deformations, the mechanical model of the bionic structure is established with the absolute node coordinate method. Utilizing a connectivity matrix to describe the connection of the bio-syncretic components, the integral form-finding method for flexible combination deformations is established and verified with FEA based on several case studies.

(3) According to the study of the mechanical properties of the bionic structure for different structural parameters, the explanation of the natural configuration as an “S” form of a bird neck is provided. In addition, the bionic structure can realize the variable dynamic stiffness property for the proposed design criteria for the QZS condition, and this result illustrates the mechanism for the bending dynamic stability of the bird neck.

Based on the structure design, mechanical model, and optimization criteria for the orientation function of the bionic rigid-flexible structure, the advantages of the bionic structure in terms of spatial accessibility and variable design for modularization are discovered. Additionally, some mechanical characteristics of the bionic structure can explain the primary biological characteristics of bird necks.

## Declaration of competing interest

The authors declare that they have no conflicts of interest in this work.

## Appendix

According to the Refs. [38], [39], [40], exhibiting the material properties of the skeletons, the elasticity modulus of the skeleton is about 2-20 GPa by material experiment of woodpecker head. The software for FEA is the ABAQUS, in which, in order to establish the rods approaching to rigid body, the elasticity modulus of the material of the rods are set as E = 20 GPa as the previous references, which is much larger than the elasticity of the bio-syncretic with the elastic stiffness coefficient *k_ik_* =10 N⋅m^−1^. The model in the ABAQUS is shown in Fig. A1.

## Appendix B Supplementary materials

Image, application 1
