# Supplementary material for: Mechanism properties of a bird-neck bionic rigid-flexible structure
Source: Fundam Res. 2022 Aug 6;4(6):1613–24. doi: 10.1016/j.fmre.2022.06.023 (PMC11670692; doi:10.1016/j.fmre.2022.06.023)
Supplement: Supplementary file 1 [file mmc1.pdf]

Certificate of English Language Editing

Manuscript Title:

Mechanism properties of a bird-neck bionic rigid-flexible structure

Date of Revision:

June 27, 2022

Abstract:

By illustrating the biological construction of a bird neck, a bionic bird-neck multilevel rigid-flexible structure is proposed and some biometric properties are explained based on the mechanism properties. The proposed structure can flexibly deform in six directions, which inspires the study of its mechanical properties for flexible deformations. First, the structural configuration and composition are determined based on the study of the anatomical characteristics of woodpeckers. Since the skeletons and muscles have very different values for the elasticity modulus and the deformation is mostly dependent on the muscle tension, the bionic structure consists of rigid units and bio-syncretic components. For combined deformations, the mechanical model is established by the connectivity matrix to describe the connection of each level. Second, based on the principle of minimum potential energy, an integral form-finding method is proposed for flexible combination deformations. All of the integral forms obtained with the theoretical analysis are compared with the results obtained with FEA. Then, the structural parameters of the...

This document certifies that the manuscript listed above was copy edited for English language by LetPub, with regard to grammar, punctuation, spelling, and clarity. All of our language editors are native English speakers with long-term experience in editing scientific and technical manuscripts. We are committed to leveling the playing field for researchers whose native language is not English.

- Documents receiving this certification should be regarded as having undergone professional editorial revision for English language before submission. However, the authors may accept or reject LetPub's suggestions and changes at their own discretion and LetPub does not have editorial control over the submitted documents.
- The language quality of the submitted document is the sole responsibility of the submitting authors subject to those authors' adherence to LetPub's revisions and instruction. LetPub's provision of service does not constitute a guarantee or endorsement of the authors' work herein.
- Neither the research content nor the authors' intended meaning were altered in any way during the editing process.
- If you have any questions or concerns about this edited document, please contact us at support@letpub.com

LetPub
Your Manuscript Editor

LetPub is an author service brand owned and operated by Accdon LLC. Headquartered in the Boston area, we are a full-spectrum author services company with a large team of US-based certified language and scientific editors, ISO 17001 accredited translators, and professional scientific illustrators and animators. We advocate ethical publication practices and are an official member of the Committee on Publication Ethics (COPE).

For more information about our company, services, and partnership programs, please visit www.letpub.com.

© 2022 Accdon, LLC. All Rights Reserved. Tel: 1-781-202-9968 Email: info@accdon.com Address: 400 Fifth Ave, Suite 530, Waltham, MA 02451, United States
